# Retraction Note: Structural relations of illness perception, fatigue, locus of control, self-efficacy, and coping strategies in patients with multiple sclerosis: a cross-sectional study

**DOI:** 10.1186/s12889-026-28125-2

**Published:** 2026-06-16

**Authors:** Amir Akbari Esfahani, Abbas Pourshahbaz, Behrooz Dolatshahi

**Affiliations:** Department of Clinical Psychology, Faculty of Behavioral Sciences, University of Social Welfare and Rehabilitation Sciences, Tehran, Iran

**Retraction Note: BMC Public Health 24, 1354 (2024)**

**https://doi.org/10.1186/s12889-024-18807-0**

The Editors have retracted this article.

After publication, concerns were raised that the Mini-Mental State Examination (MMSE) instrument was used without proper permissions. The authors were contacted, but they were unable to provide a satisfactory explanation or documentation for securing permissions.

The authors did not respond to the correspondence from the Publisher about this retraction.

